# Supplementary material for: Sub-2 Angstrom resolution structure determination using single-particle cryo-EM at 200 keV
Source: J Struct Biol X. 2020 Feb 28;4:100020. doi: 10.1016/j.yjsbx.2020.100020 (PMC7337053; doi:10.1016/j.yjsbx.2020.100020)
Supplement: Supplementary data 1 [file mmc1.docx]

| 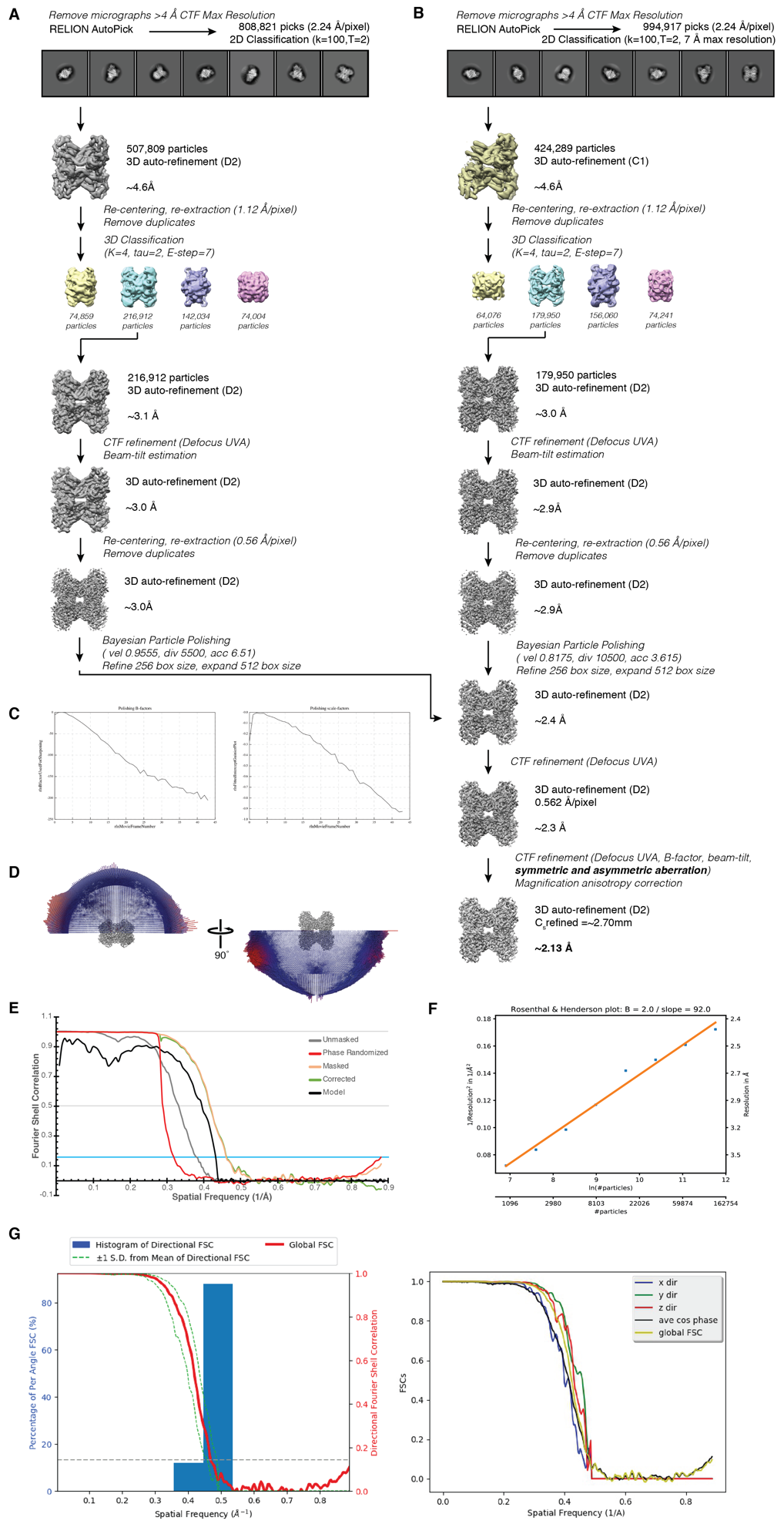 |
| --- |
| **Supplementary Figure 1. Schematic for aldolase single-particle cryo-EM data processing.** ~800K particles **(A)** and ~990K particles **(B)** were extracted from aligned, non-dose-weighted micrographs, Fourier-binned 4 x 4 (64 pixel box, 2.24 Å/pixel), and subjected to two independent rounds of reference-free 2D classification using RELION 3.0 (Zivanov et al., 2018). Representative class averages are shown. Particles comprising the “best” classes were 3D auto-refined to ~4.6 Å resolution. Particles were re-centered and re-extracted Fourier-binned 2 x 2 (128 pixel box, 1.12 Å/pixel), and subjected to 3D classification (K=4, tau_fudge=2, E-step=7 Å). ~217K **(A)** and ~180K **(B)** particles comprising the best class were selected for 3D auto-refinement followed by CTF refinement (defocus UV, whole micrograph astigmatism), including beam-tilt estimation, and another round of 3D auto-refinement. Particles were re-centered and re-extracted (256 pixel box, 0.56 Å/pixel), subjected to 3D auto-refinement, and the outputs were used for Bayesian particle polishing to yield “shiny” particles (512 pixel box, 0.56 Å/pixel). Plots of the calculated B-factors and scale factors from Bayesian particle polishing for dataset **(B)** are shown in **(C).** The outputted “shiny” particles from both datasets were combined (~390K particles) and subsequently 3D auto-refined to yield a ~2.4 Å resolution structure. CTF refinement (defocus UV, whole micrograph astigmatism), and subsequent 3D auto-refinement yielded a ~2.3 Å resolution structure. An additional round of CTF refinement including higher-order optical aberration correction and C_s_ refinement yielded a ~2.1 Å resolution reconstruction. **(D)** Plots showing the Euler angle distribution of the final aldolase EM density. **(E)** Gold-standard Fourier shell correlation (FSC) curve generated from the independent half maps contributing to the ~2.1 Å resolution aldolase EM density. FSC curve between the final refined atomic model and aldolase EM density is also shown. **(F)** Rosenthal and Henderson plot for particles contributing to the final aldolase reconstruction. **(G)** Histogram and directional 3D FSC plots (Tan et al., 2017) (sphericity = 0.974 out of 1). |

| 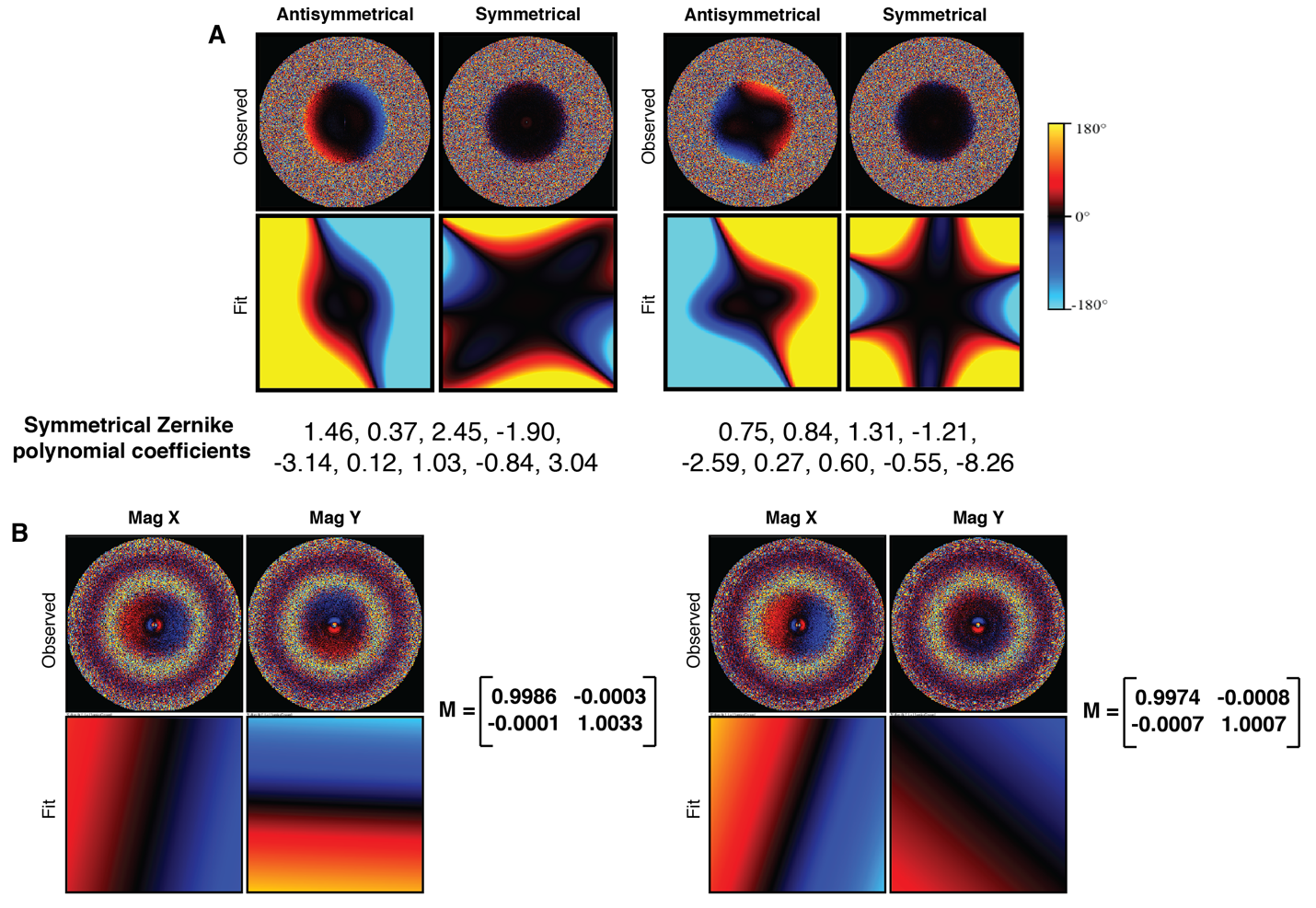 |
| --- |
| **Supplementary Figure 2. Estimated aberrations and magnification anisotropy for aldolase datasets. (A)** Anti-symmetrical and symmetrical aberrations estimated for both datasets (left, dataset corresponding to **SI Fig. 1A**; right, dataset corresponding to **SI Fig. 1B** using RELION 3.1 (Zivanov et al., 2019). Per-pixel phase-angle estimates are depicted in the upper plots, and parametric fits using Zernike polynomials are shown in the lower plots. **(B)** Magnification anisotropy estimated for both datasets. Observed and fit per-pixel displacements are depicted in the upper and lower plots, respectively, and the estimated magnification matrix *M* is included on the right. |

| 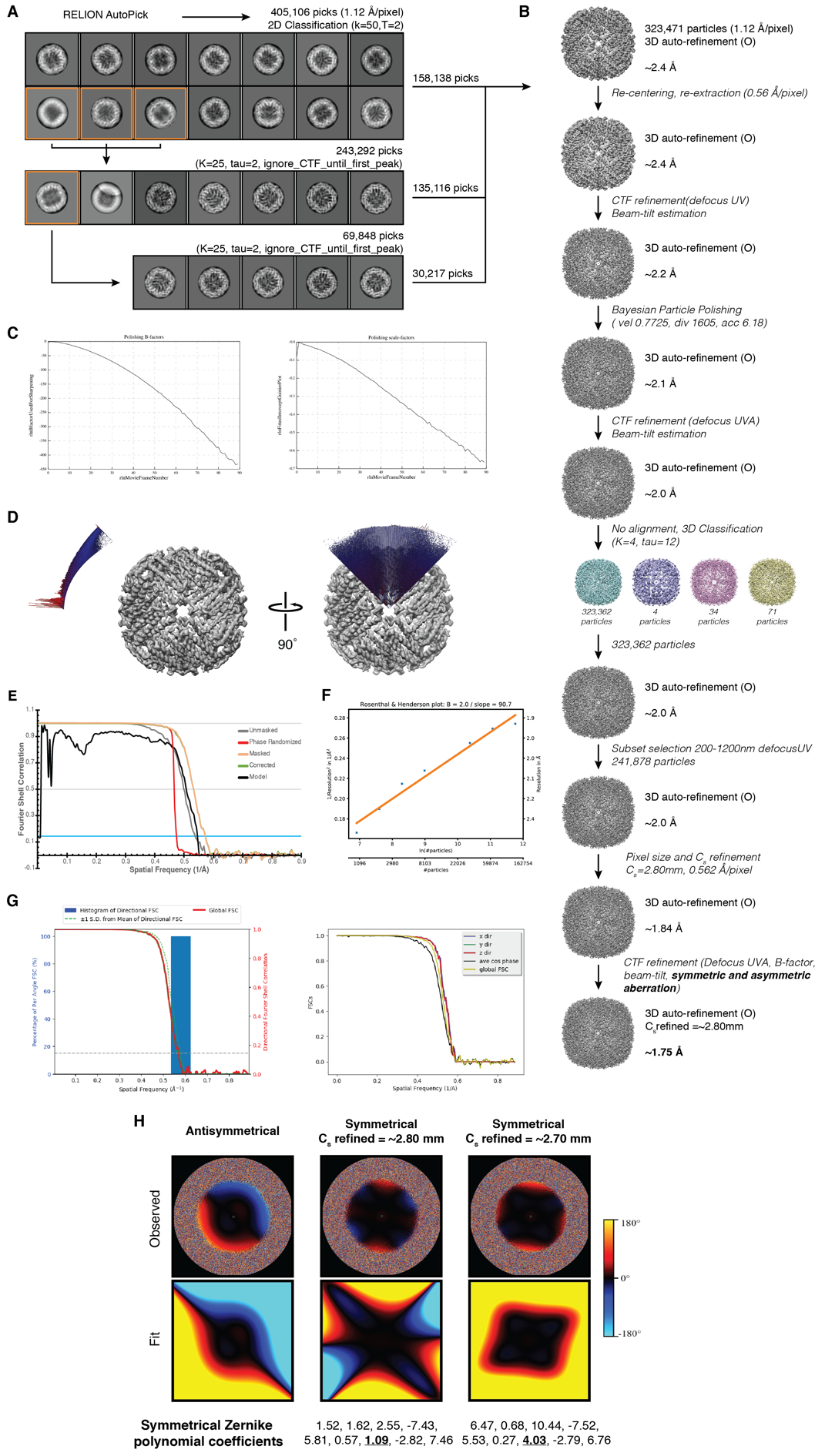 |
| --- |
| **Supplementary Figure 3. Schematic for apoferritin single-particle cryo-EM data processing.** **(A)** ~400K apoferritin particles were extracted from aligned, non-dose-weighted micrographs, Fourier-binned 2 x 2 (192 pixel box, 1.12 Å/pixel), and subjected to three subsequent rounds of reference-free 2D classification using RELION 3.0 (Zivanov et al., 2018). Representative class averages are shown. Particles comprising the “best” classes were saved while the particles contributing to class averages highlighted in orange were subjected to additional rounds of 2D classification. **(B)** These particles were combined and subsequently 3D auto-refined to ~2.4 Å resolution (O symmetry). Particles were re-centered and re-extracted unbinned (384 pixel box, 0.56 Å/pixel), and subjected to 3D auto-refinement followed by CTF refinement (defocus UV, whole micrograph astigmatism), including beam-tilt estimation. After another round of 3D auto-refinement the outputs were used for Bayesian particle polishing to yield “shiny” particles (384 pixel box, 0.56 Å/pixel). Plots of the calculated B-factors and scale factors from Bayesian particle polishing are shown in **(C).** The outputted “shiny” particles were 3D auto-refined to yield a ~2.1 Å resolution structure. CTF refinement (defocus UV, whole micrograph astigmatism), including beam-tilt estimation and subsequent 3D auto-refinement (O symmetry) yielded a ~2.0 Å resolution structure. Particles imaged between 200-1200 nm underfocus were selected (~240K particles) and 3D auto-refined to ~2 Å resolution. Changing the spherical aberration (C_s_) value to 2.80 mm and using a refined pixel size of 0.562 Å/pixel followed by CTF refinement (defocus UV, whole micrograph astigmatism) and subsequent 3D auto-refinement yielded a ~1.8 Å resolution reconstruction. An additional round of CTF refinement including higher-order optical aberration correction and C_s_ refinement yielded a ~1.7 Å resolution reconstruction. **(D)** Plots showing the Euler angle distribution of the final apoferritin EM density. **(E)** Gold-standard Fourier shell correlation (FSC) curve generated from the independent half maps contributing to the ~1.7 Å resolution apoferritin reconstruction. FSC curve between the final refined atomic model and apoferritin EM density is also shown. **(F)** Rosenthal and Henderson plot (Rosenthal and Henderson, 2003) for particles contributing to the final apoferritin reconstruction. **(G)** Histogram and directional 3D FSC plots (Tan et al., 2017) (sphericity = 0.993 out of 1). **(H)** Anti-symmetrical and symmetrical aberrations (for refined C_s_=~ 2.80 mm and ~2.70 mm) estimated using RELION 3.1 (Zivanov et al., 2019). Per-pixel phase-angle estimates are depicted in the upper plots, and parametric fits using Zernike polynomials are shown in the lower plots. |

| 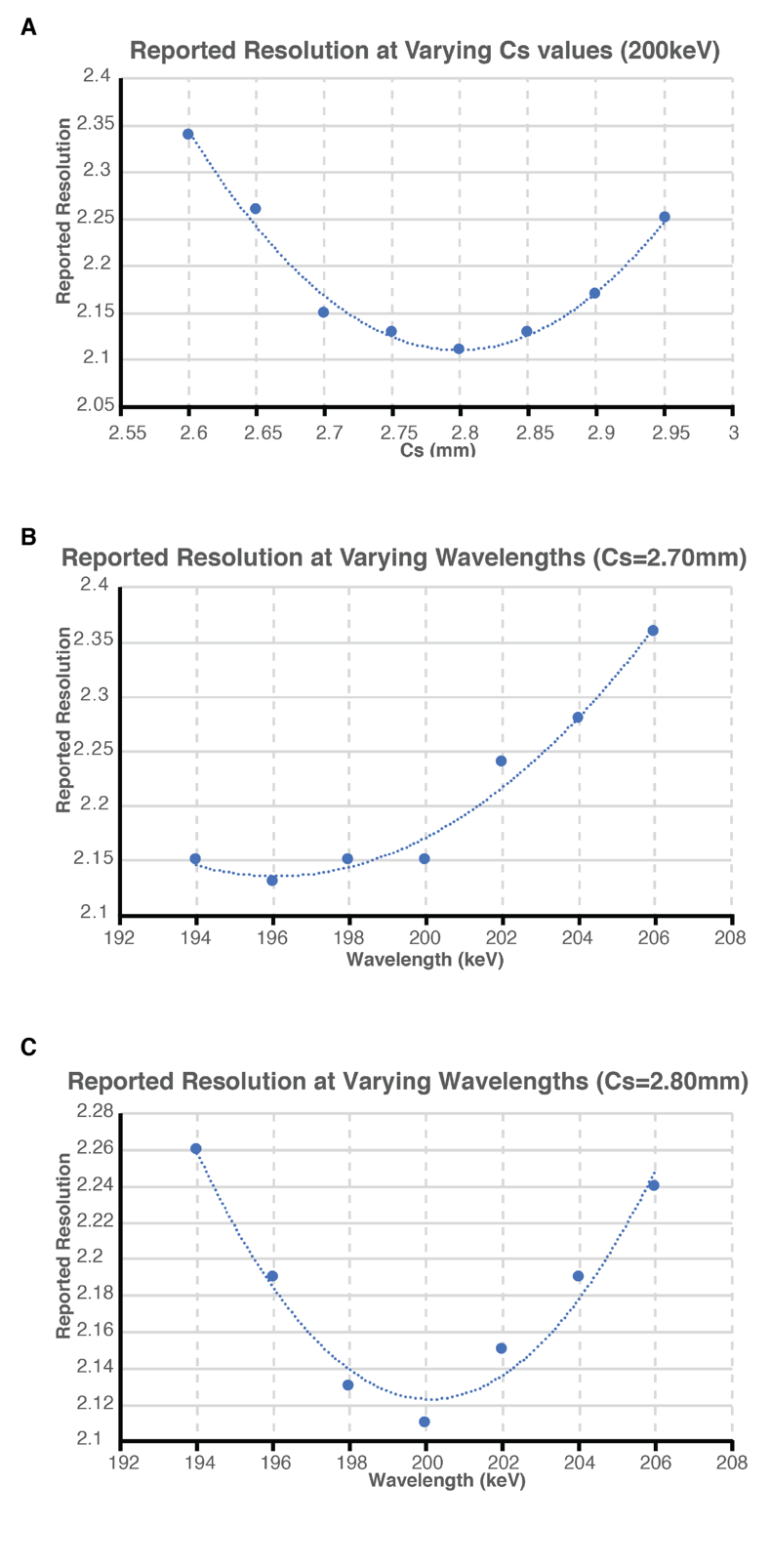 |
| --- |
| **Supplementary Figure 4. Refinement of spherical aberration and wavelength values.** **(A)** FSC-reported resolution values for a subset of the apoferritin data (~12K particles) in which the C_s_ value was manually changed prior to CTF refinement (defocus UV) and subsequent 3D auto-refinement. Wavelength (200 keV), amplitude contrast (0.1), and astigmatism were kept constant. **(B)** FSC-reported resolution for a subset of the apoferritin data (~12K particles) where the wavelength was manually changed prior to CTF refinement (defocus UV) and subsequent 3D auto-refinement. The wavelength was varied for subsets using a C_s_=2.80 mm value or **(C)** a C_s_=2.70 mm value. All 3D auto-refinements used the same parameters. |

| 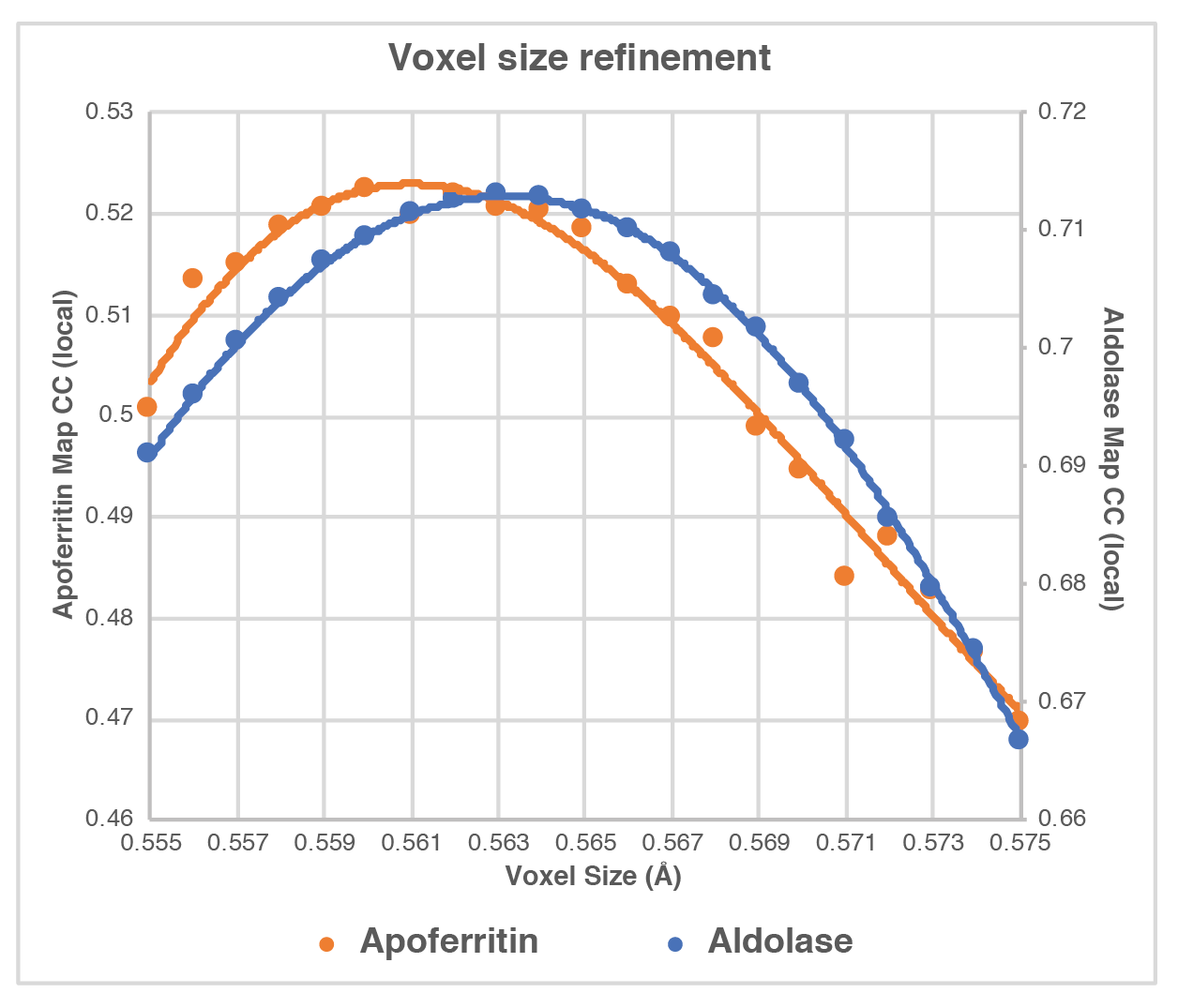 |
| --- |
| **Supplementary Figure 5. Refinement of map voxel size – aldolase and apoferritin.** Measured model­-map CC values following Phenix (Adams et al., 2010) rigid-body refinement of aldolase (blue dots, PDB ID: 5VY5) or apoferritin (orange dots, PDB ID: 3WNW) asymmetric units (ASUs) into the EM reconstructions of varying voxel size. Polynomial fits for each data set are shown as solid lines. |

**References**

Adams, P.D., Afonine, P.V., Bunkoczi, G., Chen, V.B., Davis, I.W., Echols, N., Headd, J.J., Hung, L.W., Kapral, G.J., Grosse-Kunstleve, R.W., McCoy, A.J., Moriarty, N.W., Oeffner, R., Read, R.J., Richardson, D.C., Richardson, J.S., Terwilliger, T.C., Zwart, P.H., 2010. PHENIX: a comprehensive Python-based system for macromolecular structure solution. Acta Crystallogr D Biol Crystallogr 66, 213-221.

Rosenthal, P.B., Henderson, R., 2003. Optimal determination of particle orientation, absolute hand, and contrast loss in single-particle electron cryomicroscopy. J Mol Biol 333, 721-745.

Tan, Y.Z., Baldwin, P.R., Davis, J.H., Williamson, J.R., Potter, C.S., Carragher, B., Lyumkis, D., 2017. Addressing preferred specimen orientation in single-particle cryo-EM through tilting. Nat Methods 14, 793-796.

Zivanov, J., Nakane, T., Scheres, S.H.W., 2019. Estimation of High-Order Aberrations and Anisotropic Magnification from Cryo-EM Datasets in RELION-3.1. bioRxiv, 798066.

Zivanov, J., Nakane, T., Forsberg, B.O., Kimanius, D., Hagen, W.J., Lindahl, E., Scheres, S.H., 2018. New tools for automated high-resolution cryo-EM structure determination in RELION-3. Elife 7.
